# Gene Polymorphisms of Epithelial Cell-Derived Alarmins and Their Effects on Protein Levels and Disease Severity in Patients with COVID-19

**DOI:** 10.3390/genes14091721

**Published:** 2023-08-29

**Authors:** Maral Ranjbar, Ruth P. Cusack, Christiane E. Whetstone, Shiraz Nawaz, Christopher Khoury, Jennifer Wattie, Lesley Wiltshire, Jennifer Le Roux, Eric Cheng, Thivya Srinathan, Terence Ho, Roma Sehmi, MyLinh Duong, Gail M. Gauvreau

**Affiliations:** 1 Department of Medicine, Division of Respirology, McMaster University, Hamilton, ON L8N 3Z5, Canada; ranjbm1@mcmaster.ca (M.R.); cusackruth@hotmail.com (R.P.C.); whetstoc@mcmaster.ca (C.E.W.); nawazs3@mcmaster.ca (S.N.); chris.khoury@mail.utoronto.ca (C.K.); wattiej@mcmaster.ca (J.W.); wiltshi@mcmaster.ca (L.W.); hot4@mcmaster.ca (T.H.); sehmir@mcmaster.ca (R.S.); duongmy@mcmaster.ca (M.D.); 2 Hamilton Health Sciences, Hamilton, ON L8N 3Z5, Canada; lerouxj@hhsc.ca; 3 St. Joseph’s Healthcare Hamilton, Hamilton, ON L8N 4A6, Canada; eric.sp.cheng@gmail.com (E.C.);; 4 The Research Institute of St. Joe’s Hamilton, Firestone Institute for Respiratory Health, St. Joseph’s Healthcare Hamilton, Hamilton, ON L8N 4A6, Canada; 5 Population Health Research Institute, McMaster University, Hamilton, ON L8N 3Z5, Canada

**Keywords:** COVID-19, severity, alarmins, single nucleotide polymorphisms, sex specific

## Abstract

Background: The immune response in COVID-19 is characterized by the release of alarmin cytokines, which play crucial roles in immune activation and inflammation. The interplay between these cytokines and genetic variations may influence disease severity and outcomes, while sex differences might further contribute to variations in the immune response. Methods: We measured the levels of alarmin cytokines in a cohort of COVID-19 and non-COVID-19 patients using a sensitive Meso Scale Discovery system. Additionally, we conducted an SNP analysis to identify genetic variations within the *IL-33* and *TSLP* genes. The association between these genetic variations, cytokine production, and COVID-19 severity was examined. Results: Our findings revealed elevated levels of IL-33 and IL-25 in COVID-19-positive patients compared to COVID-19-negative patients (*p* < 0.05), indicating their potential as therapeutic targets for disease modulation. Moreover, a minor allele within the IL-33 gene (rs3939286) was found to be associated with a protective effect against severe COVID-19 (*p* < 0.05), and minor alleles of the *TSLP* gene (rs2289276 and rs13806933) were found to significantly reduce TSLP protein levels in serum (*p* < 0.05). Sex-specific effects of *TSLP* and *IL-33* SNPs were observed, suggesting a potential influence of sex hormones and genetic variations on the regulation of cytokine production. Conclusion: The present study highlights the importance of alarmin cytokines and genetic variations in COVID-19 severity, providing valuable insights into personalized treatment approaches. Our results suggest that targeting alarmin cytokines may offer potential therapeutic benefits in managing COVID-19. Furthermore, the sex-specific effects of genetic variations emphasize the need to consider individual genetic profiles and sex differences when designing targeted interventions.

## 1. Introduction

COVID-19 (coronavirus disease 2019) is a respiratory disease caused by the SARS-CoV-2 virus and has a wide range of symptoms and complications. Extensive research has revealed the complex interplay of immune cell alterations, cytokine dysregulation, and genetic predisposition that play a pivotal role in the disease’s pathology [1]. Clinical features vary from asymptomatic cases to severe forms of illness leading to respiratory failure, and this variability is influenced by underlying medical comorbidities as well as genetic factors [2].

The dysregulation of cytokines is a crucial factor in the progression and severity of COVID-19. When the virus invades host cells, immune cells of the innate immune system including neutrophils, macrophages, eosinophils, and dendritic cells (DCs) migrate to the infected site and release proinflammatory cytokines such as interferons (INF), TNF (tumor necrosis factor), IL-6, IL-1, and chemokines CCL2, CCL3, and CCL4, which consequently leads to the recruitment of adaptive immune cells T cells to the inflamed area [3]. This coordinated immune response plays a key role in the pathogenesis of the disease.

An exaggerated and aberrant immune response triggers the excessive production of numerous inflammatory cytokines, creating a positive feedback loop. This cascade leads to the recruitment of additional immune cells and subsequent further cytokine production, resulting in widespread inflammation and potential organ damage [4]. This phenomenon, known as a cytokine storm, is particularly observed in more severe cases of the disease [5].

Among the different cytokines produced in response to the viral infection, epithelium derived cytokines also known as alarmin cytokines including IL-33, TSLP (thymic stromal lymphopoietin), and IL-25, are among the first proteins released from the damaged host epithelial cells and play regulatory roles in both innate and adaptive immune responses. Alarmin cytokines initiate a series of events, triggering the migration of mast cells, DCs, and T cells to the inflamed regions of the lungs, where they work together to combat the virus. Detecting these cytokines in human samples has been challenging due to their low levels, making them difficult to detect with standard commercial assays. Advanced and sensitive detection methods are needed to accurately measure these cytokines and gain a better understanding of their role in immune responses and diseases like COVID-19 [6,7].

Clinical manifestations of COVID-19 exhibit considerable variability among individuals, which can be attributed to various factors, including genetics. Genetic variations can play a role in determining the extent and nature of the immune response mounted by each patient, thereby contributing to the diversity observed in COVID-19 severity and outcomes [8]. Single nucleotide polymorphisms (SNPs) are common variations in the genome where a single nucleotide base differs between individuals. While many SNPs are harmless, those occurring within exons, introns, or promoter regions of genes can lead to alterations in gene expression or protein structure, having significant effects on protein production and function, impacting cellular processes, and changing disease risk and severity [9]. 

Previous studies have found that variations in the gene of the alarmin cytokine *TSLP* are associated with the risk of asthma [10,11,12,13]. Building upon this research, our study aims to investigate the impact of polymorphisms in the gene for *TSLP* and other alarmins on protein production and the severity of COVID-19 in affected patients, and to determine if alteration in the production of alarmins can serve as potential biomarkers for this viral respiratory infection.

## 2. Materials and Methods

### 2.1. Study Participants and Recruitment

The study was conducted following the approval of the Hamilton Integrated Research Ethics Committee. Between February and October 2021, COVID-19 patients as confirmed positive by real-time reverse-transcription-PCR (RT-PCR) were recruited upon hospital admission. Additionally, age- and sex-matched COVID-19-negative patients who were hospitalized but did not exhibit respiratory infections or respiratory tract-related diseases were included in the study as a control group. 

### 2.2. Demographics and Clinical Characteristics

Demographic information, clinical characteristics, and outcomes of the patients were extracted from the electronic patient records. We adopted a categorization method previously described by the food and drug administration [14] to develop a COVID-19 scale for classification of patients into three distinct groups; mild, moderate, and severe. The mild group consisted of patients who were not hospitalized or hospitalized without the need for oxygen therapy; the moderate group included patients that required non-invasive oxygen therapy, and the severe group included patients who experienced adverse outcomes and required both non-invasive and invasive ventilation. 

### 2.3. Blood Sampling

In COVID-19-positive patients, blood samples were obtained within 10 days of testing positive. Blood was collected into EDTA-containing vacutainers. 

Serum was evaluated for TSLP, IL-33, and IL-25 cytokine levels using an ultra-sensitive human cytokine multi-plex electrochemiluminescence-based immunoassay (Meso Scale Discovery, Rockville, MD, USA). The assay was performed using the MESO QuickPlex SQ 120 MM instrument (MSD, Gaithersburg, MD, USA) following the manufacturer’s protocol. The protein levels were quantified in picograms per milliliter of blood (pg/mL).

Genomic DNA isolation was performed using the Qiagen DNAeasy extraction kit, following the manufacturer’s protocol for whole blood samples. DNA purity and concentration were measured using a Nanodrop spectrophotometer and diluted to a concentration of 10 ng/μL for optimal genotyping results. For genotyping analysis of single nucleotide polymorphisms (SNPs) in the *TSLP* gene (rs2289267, rs1837253, and rs3860933) and *IL-33* gene (rs3939286 and rs1342326), the TaqMan^®^ SNP genotyping assay kit (Applied Biosystems; Foster City, CA, USA) was utilized. The assay was carried out in a 25 μL reaction volume, containing 12.50 μL of TaqMan genotyping master mix from Thermo Fisher Scientific, Waltham, MA, USA, 1.25 μL TaqMan genotyping assay mix (20×), and 11.25 μL DNA template. The thermal cycling conditions included an initial enzyme activation step at 95 °C for 10 min, followed by 40 cycles of 95 °C for 15 s and 60 °C for 1 min. The genotyping data were tested for Hardy–Weinberg equilibrium using Pearson’s chi-square test.

### 2.4. Statistical Analysis

The data are presented as mean ± standard deviation (SD). A significance level (α) of 0.05 was used for statistical analysis. Data analysis was conducted using IBM SPSS Advanced Statistics (IBM Corp., Version 24.0, Armonk, NY, USA) and GraphPad Prism software version 9.0 (GraphPad Software, San Diego, CA, USA). Independent *t*-tests and analysis of variance (ANOVA) and their non-parametric equivalents for data not normally distributed were performed to assess differences between groups. To investigate associations between individual polymorphisms and COVID-19 severity, multinomial regression analysis was employed, and odds ratios (OR) along with their 95% confidence intervals (CI) are reported.

## 3. Results

### 3.1. Patient Characteristics

In total, 84 COVID-19-positive and 65 COVID-19-negative age- and sex-matched patients were recruited (Table 1). Of the 84 COVID-19-positive patients, 54 (77.1%) received steroid treatment during their hospitalization, whereas only 5 (9.6%) COVID-19-negative patients were treated with steroids. The COVID-19-negative group had a higher frequency of smokers and a higher prevalence of comorbidities hypertension, dyslipidemia, and osteoarthritis (*p* < 0.05). Among patients, the proportion of non-smokers was significantly greater in the COVID-19-positive group in comparison to the COVID-19-negative group (*p* < 0.05).

### 3.2. Alarmin Cytokine Levels 

**Figure 1:**
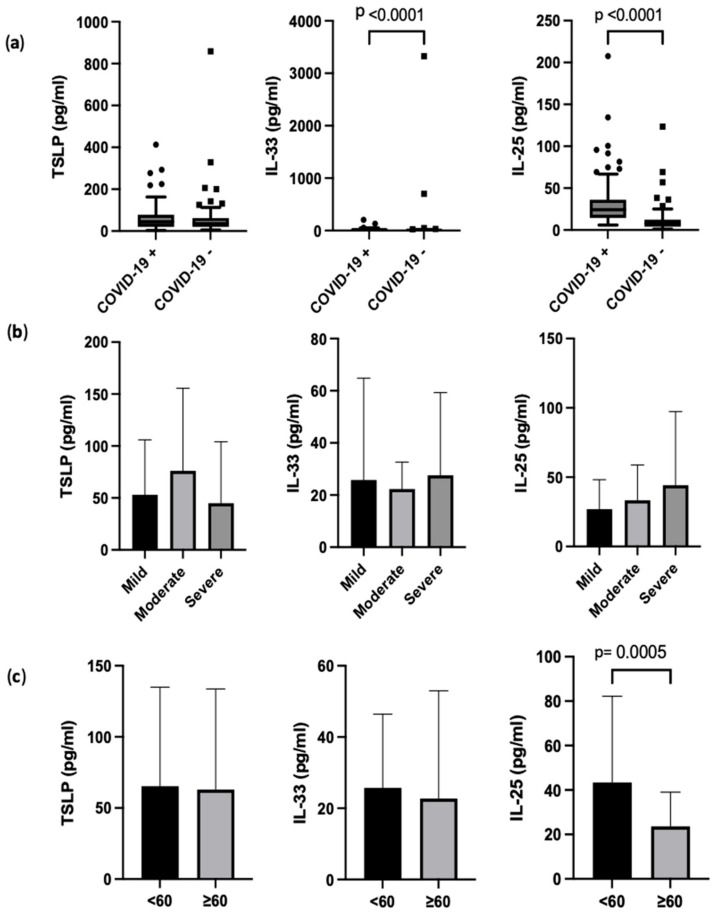
Comparison of alarmin cytokine levels in (**a**) COVID-19-positive and COVID-19-negative patients, (**b**) COVID-19-positive patients divided into mild, moderate, and severe intensity with steroid treatment in 8/19, 39/43, and 7/8, respectively, and (**c**) COVID-19-positive patients divided by age (above or below 60 years old). Statistical testing was conducted using independent *t* test and one-way ANOVA. COVID-19-negative patients had significantly lower levels of IL-33 and IL-25 (*p* < 0.05) (**a**). There was no difference in the level of alarmins between the groups regarding different severities (**b**). When stratified based on age, COVID-19-positive patients younger than 60 years old were shown to have lower levels of IL-25 (**c**).

The concentration of alarmin cytokines IL-33, TSLP, and IL-25 were compared in COVID-19-positive and -negative patients, and the association of alarmin cytokine levels with disease severity and age-related variations was evaluated (Figure 1). Significantly higher levels of total IL-33 and IL-25 were measured in the serum of COVID-19-positive patients compared to COVID-19-negative individuals (Figure 1a). TSLP levels were not significantly different, though numerically higher in the COVID-19-positive group (*p* = 0.635).

Alarmin cytokine levels compared across different severities of COVID-19-positive patients, categorized based on their oxygen requirements and ventilation routes, showed no significant difference (Figure 1b). Across the different severities, 8 out of 19 (42.1%), 39 out of 43 (90.7%), and 7 out of 8 (87.5%) patients were receiving steroid treatment in the mild, moderate, and severe categories, respectively. To explore the impact of age, we conducted a comparative analysis of alarmin protein levels between individuals above and below the mean age of 60 years. IL-25 levels were significantly lower in patients older than 60 years (*p* < 0.05), indicating an age-related variation in this cytokine (Figure 1c).

### 3.3. Genetic Variation of TSLP and IL-33 Genes and Effect on Disease Severity

A TaqMan SNP genotyping assay was employed to determine the frequency of SNP genotypes for *TSLP* (rs2289276, rs1837253, and rs3806933) and *IL-33* (rs3939286 and rs1342326). The frequency of homozygous major, heterozygous, and homozygous minor genotypes for these SNPs was not different when comparing the COVID-19-positive to COVID-19-negative patients (Table 2). Among the genotypes, the homozygous major genotype exhibited the highest prevalence among patients, while the homozygous minor genotype showed the lowest prevalence, confirming that the distribution of SNP genotypes is similar between COVID-19-positive and COVID-19-negative individuals, with the homozygous major genotype being the most common and homozygous genotype being the least common among the studied patients.

We conducted a multinomial regression analysis to explore potential relationships between *TSLP* and *IL-33* SNPs and COVID-19 severity (Table 2). Among the *TSLP* SNPs, we did not find any significant associations with COVID-19 severity; however, for *IL-33*, the homozygous minor genotype of rs3939286 demonstrated a significant association with the severe form of the disease (*p* = 0.033) with an odds ratio of 0.109 (95% confidence interval 0.14–0.839) indicating an inverse association. These results suggest that individuals carrying the minor genotype of rs3939286 have a protective effect against severe COVID-19 outcomes. In practical terms, this indicates that the presence of the minor genotype of rs3939286 may offer some level of protection to COVID-19-positive patients, reducing the likelihood of developing severe forms of the disease. The protective effect of the minor allele (T) of rs3939286 was consistently seen in all carriers (CT + TT), showing a strong association with reduced severity of COVID-19 (*p* = 0.045, OR = 0.131, CI = 0.018–0.951).

### 3.4. The Effect of TSLP and IL-33 SNPs on Protein Levels

**Figure 2:**
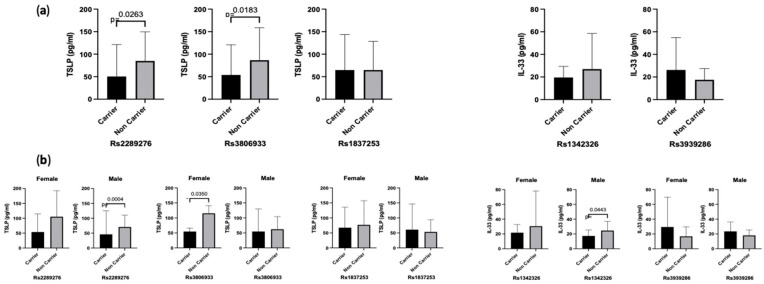
The effect of single nucleotide polymorphisms (SNPs) on (**a**) serum levels, and (**b**) and sex-specific serum levels of TSLP and IL-33 in COVID-19-positive patients. Carriers of *TSLP* SNPs rs2289276 and rs3806933 showed lower levels of TSLP cytokine in their serum indicating a protective pattern of these SNPs. *IL-33* SNPs, however, did not show any protective nor direct associations (**a**). When stratified for gender, male carriers of Rs2289276 and female carriers of Rs3806933 showed lower TSLP levels. Male carriers of Rs1342326 also showed lower IL-33 levels (**b**).

Regression analysis of *TSLP* and *IL-33* SNPs revealed significant relationships between certain genetic variants and the production of TSLP and IL-33 proteins (Figure 2a). Carriers of the minor allele (CT + TT) for *TSLP* SNPs rs2289276 and rs3806933 showed lower serum levels of TSLP protein compared to non-carriers (*p* = 0.0263 and *p* = 0.0183, respectively) demonstrating the mutant genotype leads to reduced protein levels. Further analysis based on gender showed sex-specific effects. Compared to non-carriers, males with the rs2289276 mutant allele and females with the rs3806933 mutant allele had significantly lower TSLP levels (*p* = 0.0004 and *p* = 0.035, respectively). Among the *IL-33* SNPs, male carriers of the rs1342326 minor allele displayed significantly lower IL-33 levels compared to non-carriers (*p* = 0.0443), while no such difference was seen in females (*p* = 0.4212). Following the initial analysis, we conducted further tests to investigate whether the protective effects of these genotypes would be evident across different severities of the disease; however, we did not observe any significant relationship. One plausible reason for this lack of significance could be attributed to lower sample numbers within each severity category. These findings indicate that certain genetic variants in *TSLP* and *IL-33* may influence protein production differently in males and females, suggesting potential sex-specific effects on disease outcomes.

## 4. Discussion

In this study, we observed a higher prevalence of non-smokers among COVID-19-positive patients in contrast to COVID-19-negative patients. Similar findings have been documented in other observational studies [15]. Although cigarette smoke has been found to disturb the innate and adaptive immunological balance [16], it is worth noting that research has demonstrated contrasting effects of nicotine. Specifically, nicotine has been linked to an increased expression of ACE2 receptors, along with the suppression of inflammatory reactions in COVID-19 cases, resulting in a dampened cytokine storm response [17]. However, it is important to acknowledge the presence of discrepancies in these findings, as certain studies have indicated an association between smoking and heightened COVID-19 severity and mortality rates [18].

We also observed significantly elevated levels of IL-33 and IL-25 proteins in COVID-19 patients compared to non-COVID individuals. These alarmin cytokines, released from damaged epithelial cells, play a crucial role in signaling danger to the immune system during viral infections. Interestingly, immune cells at inflamed sites also contributed to the production of these cytokines, suggesting a broader involvement beyond the epithelium [4]. The sensitive Meso Scale Discovery system was essential for accurately detecting these cytokines due to their low concentrations in blood. The crucial significance of alarmin cytokines in respiratory diseases has been established. These cytokines play a substantial role in shaping the immune response and regulating inflammation within the airways. Emitted in reaction to various environmental stimuli, they serve as vital early warning signals, prompting the activation of both innate and adaptive immune responses [19,20,21]. Therefore, our current study focuses on the pivotal role of alarmin cytokines as central drivers of immune processes, particularly in the context of respiratory infections.

Maintaining a delicate balance of cytokine responses is vital for an effective immune defense against pathogens. Overproduction of alarmin cytokines may lead to dysregulated immune responses and exacerbate the pathophysiological consequences of the infection. Studies have indicated that blocking IL-25 can enhance antiviral immune activity [22]. Additionally, IL-33 has been linked to lung injury, pulmonary viral infections, and chronic lung diseases, indicating a potential association between elevated IL-33 levels and the severity of respiratory manifestations observed in COVID-19 patients [23,24]. While TSLP showed a numerical increase in COVID-19 patients, statistical significance was not achieved in our study due to the low sample size. Nevertheless, previous research has reported a correlation between TSLP levels and the duration of hospitalization in COVID-19 patients, suggesting a possible role of TSLP in disease progression [7]. 

IL-33, TSLP, and IL-25 have been implicated as potential predictors of severe COVID-19 outcomes, associated with severe lung inflammation, making them promising candidates for disease severity control [25,26]. However, our study did not find significant variations in alarmin cytokine levels across different disease severities. This lack of significant differences could be attributed to the widespread use of steroid treatment among a considerable number of patients during our recruitment phase. Steroids have well-known immunomodulatory effects, potentially influencing cytokine production and the immune response [27]. When we examined blood drawn within 7 days of testing positive for COVID-19, we found higher IL-25 levels compared to blood collected after 7 days (39.14 ± 36.44 vs. 26.15 ± 20.31); however, we did not find any significant differences in IL-33 and TSLP levels between the two different blood draw groups (*p* > 0.05). These findings suggest a potential effect of a longer steroid treatment period on IL-25 levels, indicating steroid treatment can modulate cytokine responses in COVID-19 patients.

The downstream pathways of IL-33 and TSLP have been shown to facilitate the recruitment of various immune cells and induce inflammation [28]. In light of this, we sought to investigate whether genetic polymorphisms could potentially modulate this immune response and influence disease outcomes. Focusing on the single nucleotide polymorphisms (SNPs) of *IL-33*, we discovered a significant inverse association between *IL-33* SNP rs3939286 and COVID-19 severity, which meant that carriers of the minor genotype were found to be protected from developing more severe forms of COVID-19. While the role of *IL-33* SNPs in viral infections, particularly COVID-19, remains largely unexplored, previous studies have indicated a protective effect of this SNP in other diseases, such as rheumatoid arthritis and cardiovascular conditions [29,30]. It is plausible to suggest that rs3939286 may play a role in regulating IL-33 production and, subsequently, lowering the risk of developing severe disease in these conditions.

The prevalence of SNPs in the general population can vary depending on the specific SNP being considered and the demographic characteristics of the population studied. Different databases with large sample sizes such as α and TOPMED provide valuable resources regarding the prevalence of different SNPs in diverse populations. Data from these sources indicate that the prevalence of *TSLP* SNPs, namely rs2289276, rs3806933, and rs1837253, in the general population is approximately 29%, 42%, and 26%, respectively. Furthermore, for *IL-33* SNPs, the prevalence of rs1342326 and rs3939286 stands at 21% and 31%, respectively. These prevalence figures underscore the dynamic nature of genetic diversity across different genomic loci and populations [31].

Following our investigation into the effects of *IL-33* SNPs on disease severity, we proceeded to explore the potential association of IL-33 and TSLP SNPs with protein production. Our analysis revealed that individuals carrying mutant alleles (CT + TT) of *TSLP* SNPs rs2289276 and rs3806033 exhibited significantly lower levels of serum TSLP. However, this reduced level was not observed in the case of rs1837253. This decrease in TSLP levels among minor genotypes could potentially influence disease severity and may offer protection against more severe outcomes. Nevertheless, our analysis did not establish a direct correlation, which could be attributed to the smaller sample size within each severity group.

The plausible explanation for the lower TSLP levels in these specific SNPs lies in their location on the *TSLP* gene’s promoter region. This region determines the fate of transcription and subsequent protein production. It is reasonable to speculate that the position of these SNPs on the promoter might play a regulatory role in TSLP protein production [32,33]. Additionally, there is evidence of two distinct isoforms of TSLP: the long isoform, which is inflammatory and easily detected, and the short isoform, with a homeostatic function that remains undetectable on a protein level by commercially available detection kits [34]. Therefore, the effect of the identified *TSLP* SNPs on the production of each isoform remains an unexplored area in our study.

After stratifying our analysis based on sex, we found that the effects of the identified *TSLP* and *IL-33* SNPs on protein production were sex specific. Specifically, in males, carriers of the rs2289276 mutant allele had significantly lower TSLP levels compared to non-carriers. Similarly, in females, carriers of the rs3806933 mutant allele exhibited lower TSLP levels compared to non-carriers. However, we did not observe significant differences in TSLP levels for the rs1837253 SNP in either sex. Additionally, among the *IL-33* SNPs, we found a significant association between the rs1342326 minor allele and lower IL-33 levels in males, while no such association was observed in females. These sex-specific findings indicate that the genetic influence on alarmin cytokine production is different between males and females. The sex-specific pattern of these SNPs has been previously reported in other diseases [10,12,35]. The underlying reasons for these sex-related disparities are not yet fully understood and require further investigation. Sex hormones and genetic variations on sex chromosomes are among the potential factors that may contribute to these sex-specific effects because estrogen and testosterone play a key role in modulating gene expression. These hormones can interact with specific gene promoters and enhancers, influencing the activity of certain genes and affecting the production of proteins, including cytokines [36].

This study provides novel insights into the role of alarmin cytokines, specifically IL-33, IL-25, and TSLP, in COVID-19. By utilizing a sensitive and advanced Meso Scale Discovery system, we were able to detect and quantify these cytokines accurately, despite their low levels in blood, offering valuable data for understanding the immune response in COVID-19 patients. We explored the potential associations between genetic polymorphisms in *IL-33* and *TSLP* and COVID-19 outcomes. The identification of a significant inverse association between *IL-33* SNP rs3939286 and COVID-19 severity suggests a potential genetic influence on disease outcomes, supporting the emerging evidence that human genetics may play a pivotal role in governing the clinical manifestations of SARS-CoV-2 infection which may have implications for risk stratification and personalized treatment approaches.

The limitations in this study include the relatively small sample size, which may have impacted the statistical power and ability to detect significant differences in some analyses. A larger cohort with diverse demographic characteristics could provide more robust and generalizable findings. While we observed associations between SNPs and cytokine levels, the study lacks functional data to elucidate the mechanisms driving these genetic influences. Further functional studies are needed to understand the underlying biology and pathways affected by these genetic variations. Future investigations could focus on developing targeted therapies that specifically regulate alarmin cytokine levels to mitigate the inflammatory response in severe cases. The sex-specific effects observed in this study underscore the importance of considering gender differences in disease pathogenesis and treatment. Integrating genetic information and sex-specific analyses could pave the way for personalized medicine approaches, tailoring treatments based on individual genetic profiles and sex-related factors.

Overall, our findings suggest that alarmin cytokines (IL-33, TSLP, and IL-25) could serve as potential therapeutic targets to modulate disease severity in COVID-19, and genetic variations influencing protein production can also protect against developing severe COVID-19.

## Figures and Tables

**Table 1:** Comparison of clinical and demographic characteristics shown as mean (SD) for numerical data and number (%) for categorical data of COVID-19-positive and COVID-19-negative patients. ** *p* < 0.005; * *p* < 0.05 significant difference between groups.

Demographics		COVID-19 Positive (N = 84)	COVID-19 Negative (N = 65)
Sex	Male	46 (54.8)	33 (49.3)
	Female	38 (45.2)	34 (50.7)
Age		60.38 (18.10)	62.53 (15.17)
Steroid Treatment		54 (77.1)	5 (9.6) **
Smoking Status			
	Never	50 (64.9)	26 (46.4) *
	Ex-smoker	19 (24.7)	18 (32.1)
	Current Smoker	8 (10.4)	12 (21.4)
Comorbidities			
Asthma		8 (9.5)	8 (11.6)
COPD		8 (9.5)	11 (15.9)
Hypertension		40 (54.1)	39 (75) *
Dyslipidemia		29 (39.7)	30 (57.7) *
Diabetes Mellitus		28 (33.3)	22 (31.9)
Atrial fibrillation		10 (14.1)	10 (19.2)
Coronary Artery Disease		13 (18.3)	12 (23.1)
Chronic Kidney Disease		6 (8.5)	9 (17.3)
GERD		22 (31.4)	19 (36.5)
Osteoarthritis		5 (7.2)	19 (38.8) *
Osteoporosis		6 (9.0)	10 (24.0)

**Table 2:** Comparison of *TSLP* and *IL-33* gene single nucleotide polymorphisms in COVID-19-positive patients across disease severity.

SNPs	Genotypes			Population			Multinomial Regression	
		COVID-19 Positive (N = 84)	COVID-19 Negative (N = 65)	Mild (N = 25)	Moderate (N = 45)	Severe (N = 14)	Moderate vs. Mild	Severe vs. Mild
							OR (95% CI)	OR (95% CI)
*TSLP*								
rs2289276	CC ^a^	34 (41)	28 (42.4)	9 (36)	20 (44.4)	5 (38.5)		
	CT	41 (49.4)	34 (51.5)	13 (52)	21 (46.7)	7 (53.8)	0.97 (0.15–6.09)	1.01 (0.07–4.25)
	TT	8 (9.6)	4 (6.1)	3 (12)	4 (8.9)	1 (7.7)	0.81 (0.26–2.59)	0.59 (0.10–3.42)
	CT + TT	49 (59)	38 (57.6)	16 (64)	25 (55.6)	8 (61.5)	0.84 (0.28–2.49)	0.66 (0.12–3.41)
rs1837253	CC ^a^	45 (54.9)	36 (55.4)	14 (58.3)	24 (53.3)	7 (53.8)		
	CT	37 (45.1)	27 (41.5)	10 (41.7)	21 (46.7)	6 (46.2)	1.05 (0.35–3.14)	0.72 (0.13–3.89)
	TT	0 (0)	2 (3.1)					
	CT + TT	37 (45.1)	29 (44.6)					
rs3806933	CC	26 (31.7)	18 (26.1)	6 (25)	15 (33.3)	5 (38.5)		
	CT	42 (51.2)	27 (39.1)	16 (66.7)	21 (46.7)	5 (38.5)	2.46 (0.35–16.99)	2.42 (0.20–28.71)
	TT	14 (17.1)	24 (34.8)	2 (8.3)	9 (20)	3 (23.1)	0.64 (0.18–2.21)	0.44 (0.68–2.95)
	CT + TT	56 (68.3)	51 (73.9)	18 (75)	30 (66.7)	8 (61.5)	1.059 (0.35–3.14)	0.72 (0.13–3.89)
*IL-33*								
rs3939286	CC ^a^	20 (23.8)	17 (24.6)	3 (12)	10 (22.2)	7 (50)		
	CT	58 (69)	48 (69.6)	21 (84)	31 (68.9)	6 (42.9)	0.76 (0.05–11.46)	0.49 (0.01–12.68)
	TT	6 (7.1)	4 (5.8)	1 (4)	4 (8.9)	1 (7.1)	0.356 (0.07–1.11)	0.10 (0.14–0.83) *
	CT + TT	64 (76.2)	52 (75.4)	22 (88)	35 (77.8)	7 (50)	0.38 (0.07–2.02)	0.13(0.01–0.95) *
rs1342326	AA ^a^	53 (63.1)	42 (60.9)	15 (60)	29 (64.4)	9 (64.3)		
	AC	11 (13.1)	11 (15.9)	4 (16)	5 (11.1)	2 (14.3)	0.96 (0.25–3.63)	0.86 (0.12–6.35)
	CC	20 (23.8)	16 (23.2)	6 (24)	11 (24.4)	3 (21.4)	0.52 (0.11–2.43)	0.513 (0.046–5.77)
	AC + CC	31 (36.9)	27 (39.1)	10 (40)	16 (35.6)	5 (35.7)	0.75 (0.24–2.31)	0.70 (0.13–3.69)

^a^ = reference genotype; OR= odds ratio. * *p* < 0.05 significant difference between groups.

## Data Availability

Data supporting reported results can be accessed by contacting the corresponding author.
